# Evaluation of the effect of metal stents on dose perturbation in the carbon beam irradiation field

**DOI:** 10.1002/acm2.70042

**Published:** 2025-02-19

**Authors:** Yuya Miyasaka, Tetsuya Ishizawa, Yoshihito Nawa, Hikaru Souda, Shohei Kawashiro, Hongbo Chai, Miyu Ishizawa, Hiraku Sato, Takeo Iwai

**Affiliations:** ^1^ Department of Heavy Particle Medical Science Yamagata University Graduate School of Medical Science Yamagata Japan; ^2^ Department of Gastroenterology Yamagata University Faculty of Medicine Yamagata Japan; ^3^ Department of Radiation Oncology Kanagawa Cancer Center Yokohama Japan; ^4^ Department of Radiology Yamagata University Faculty of Medicine Yamagata Japan

**Keywords:** bile duct stent, carbon‐ion therapy, dose measurement, metal stent, pancreas cancer

## Abstract

**Propose:**

Carbon ion therapy is indicated for cases in which stents have been inserted, such as bile ducts, but the effect of metal stents on carbon ion therapy is unclear. In this study, the dose perturbation of carbon ion therapy caused by metallic bile duct stents was evaluated by dosimetry.

**Materials and methods:**

Five different types of metal stents (EGIS Double Bear Biliary Stent, EGIS Single Bear Covered Biliary Stent, BileRush Selective, Niti‐S Less Shortening D‐type Stent, and ZEO Stent V) were placed between solid phantoms and Gafchromic film were placed around the stents and at each depth. The phantom was irradiated with a Carbon ion Beam to form a 5 cm × 5 cm × 5 cm spread‐out Brag peak. The dose change due to the stent was evaluated by comparing the film values of similar irradiations without the stent in place.

**Result:**

The results showed that the dose perturbation with and without the stent was <10%. A dose reduction area along the shape of the stent appeared at the end of the irradiation field.

**Conclusion:**

The effect of metal stents on the carbon dose distribution was assumed to be comparable to that of x‐rays and protons.

## 1 INTRODUCTION

Pancreatic cancer is associated with a poor prognosis.^1^ Hence, it is important to further improve treatment outcomes. Among the various treatment options for pancreatic cancer, carbon ion radiotherapy (CIRT) has recently been found to have good outcomes.^2^, ^3^, ^4^, ^5^ CIRT, which can be applied to inoperable or recurrent cases, plays an extremely important role in pancreatic cancer management.

In the course of pancreatic cancer, the bile duct can be obstructed by the tumor. Previous reports revealed that 70% of patients with pancreatic cancer had bile duct obstruction.^6^, ^7^ To address this issue, stents are often inserted into the bile duct to ensure patency. The two main types of stent materials are metal and plastic. Based on different studies and reviews, metal stent insertion has higher patency rates and lower reintervention rates than plastic stent insertion.^8^, ^9^, ^10^, ^11^, ^12^, ^13^ Based on this finding, rather than plastic stent insertion, metal stent insertion, is preferred in numerous cases. Radiotherapy is considered for palliation of bile duct obstruction in patients with pancreatic cancer who had stent insertion. However, patients with metal stents in the ductal organ who are receiving radiotherapy were previously found to be at risk of major ductal organ adverse events.^14^, ^15^, ^16^ To prevent severe side effects, the metal stent was often replaced with a plastic stent before the start of irradiation.^17^ Alternatively, cases with metal stent insertion were excluded from the indication in cases of particle therapy for pancreatic cancer.^5^, ^18^ Nevertheless, only a few reports have shown that the use of metal stents and particle therapy are associated with adverse events, and the causal relationship between them is not clearly elucidated. In addition, most reports on adverse events related to x‐ray radiation therapy were published in the 2000s and early 2010s.^14^, ^19^, ^20^ Stent materials and weaving methods have evolved over recent years, thereby making it easier for stents to follow the shape of the lumen and retain their shape.^21^ Moreover, a recent study on the validation of the treatment technique has reported that radiotherapy may be safely provided to patients with metal stents.^22^, ^23^ However, the causes of serious adverse events in patients with metal stents receiving CIRT have not been clearly elucidated. Therefore, to identify the causes, further studies must be conducted. In particular, changes in dose beyond what is expected can be a major factor associated with adverse events and, thus, should be considered. The purpose of this study is to evaluate the dose perturbation around the metal stent caused by the interaction between the metal stent and the carbon ion beam. We then evaluated the dose perturbation of the carbon ion beam in comparison with previously reported x‐ray and proton beams. The dose perturbation was comparable to that of x‐rays and proton beams, which have been reported previously. Based on the basic studies in this study, it is desirable to further evaluate the impact of metal stents in carbon beam therapy in more detail through Monte Carlo simulations and experiments that reproduce clinical situations.

## 2 MATERIAL AND METHODS

### 2.1 Measurement geometry

**FIGURE 1:**
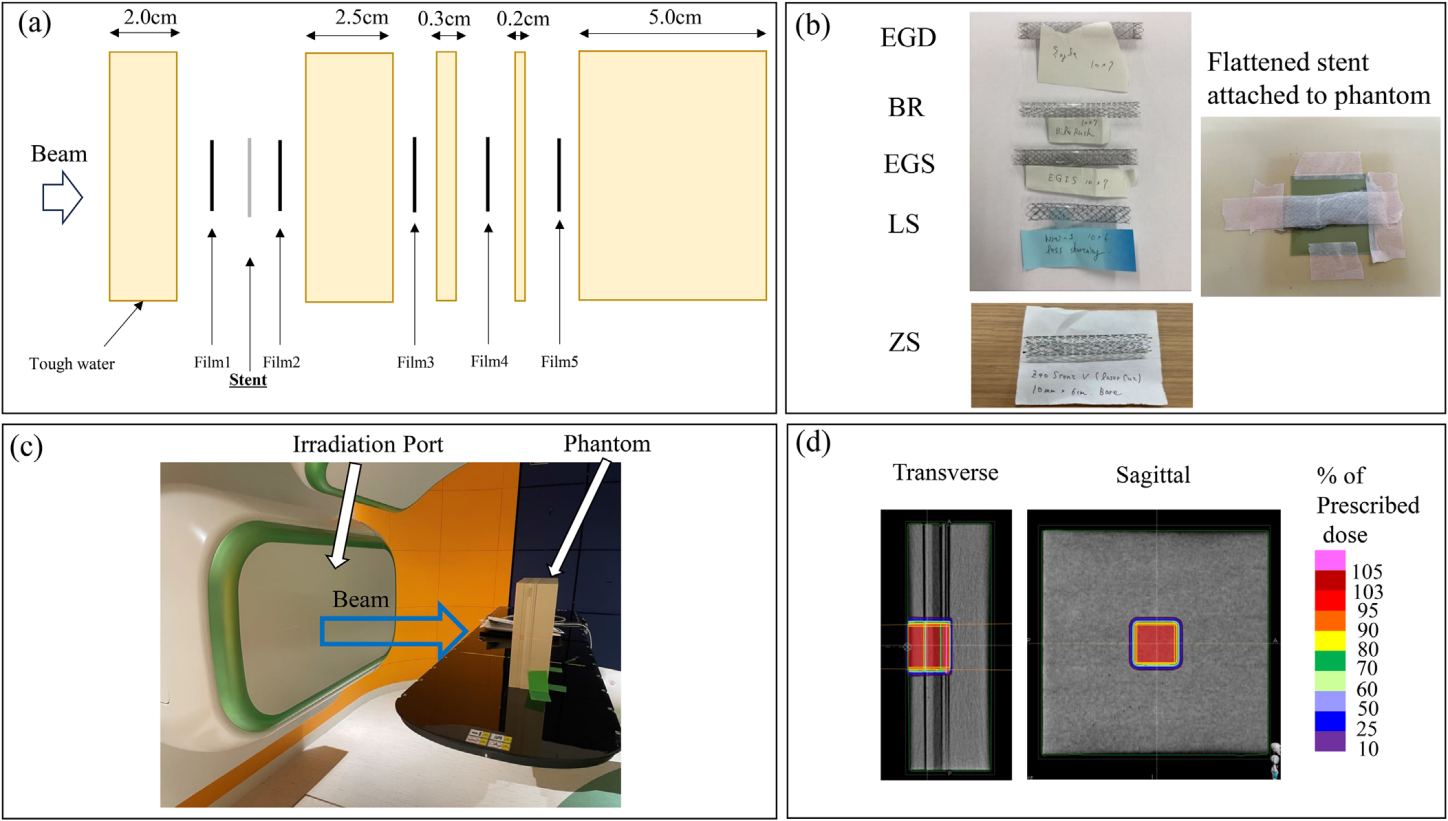
Measurement conditions. (a) geometry of the phantom and location of the stent and film, (b) five metal stents, (c) irradiated port phantom, and (d) irradiated dose distribution. BR, BileRush Selective; EGD, EGIS biliary stent double bear; EGS, EGIS biliary stent single bear covered; LS, Niti‐S Less Shortening D‐type Stent; ZS: ZEO Stent V.

A water equivalent solid phantom (Tough Water, Kyoto Kagaku Co., Ltd., Kyoto, Japan) was used as the phantom (Mass density: 1.01 g/cm^3^, Effective anatomic number: 7.96, Electron density 3.25 ×10e^26^/kg, Elemental composition: H, C, N, O, Cl, Ca). Gafchromic EBT4 (Ashland, Bridgewater, NJ, the USA, Lot#04072303) resized to 5.0 × 5.0 cm was sandwiched at each depth position in the solid phantom from the beam incidence side (Figure 1a). One of these flattened stents was placed between the films at a depth of 2.0 cm from the phantom surface. Figure 1b shows the five types of stents used for validation in this study. The stents used were EGIS Double Bear Biliary Stent (SB‐Kawasumi Laboratories Inc., Kawasaki, Japan), BileRush Selective (Piolax Inc., Yokohama, Japan) (BR), EGIS Single Bear Covered Biliary Stent (SB‐Kawasumi Laboratories Inc., Kawasaki, Japan), Niti‐S Less Shortening D‐type Stent (Taewoong Medical, Seoul, Korea), and ZEO Stent V (Zeon Medical Inc., Tokyo, Japan). The materials and formation methods of each stent were not shown in this paper because they are confidential to each vendor and not publicly available. A phantom with a film and stent sandwiched between it was placed opposite the CIRT irradiation port (Figure 1c).

### 2.2 Irradiation setting

**FIGURE 2:**
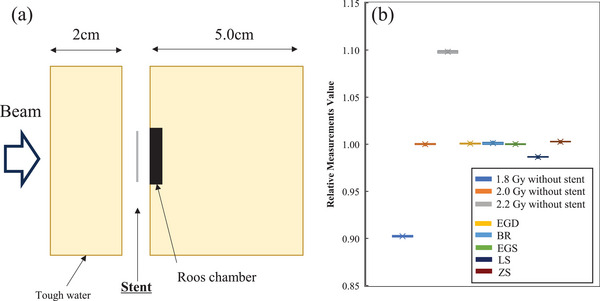
Measured geometry (a) and results (b) using an ionization chamber. BR, BileRush Selective; EGD, EGIS biliary stent double bear; EGS, EGIS biliary stent single bear covered; LS, Niti‐S Less Shortening D‐type Stent; ZS, ZEO Stent V.

First, to confirm the response of the carbon ion beam to the EBT4 film, the carbon ion beam was irradiated in a situation similar to the geometry shown in Figure 1a, but without the metal stent. The dose in the volume of interest (VOI) was irradiated to 0.5, 1.0, 1.5, and 2.0 Gy, respectively. The size of the VOI was 5.0 × 5.0 × 5.0 cm. The computed tomography scan images were acquired with the film sandwiched between phantoms. Aquilion ONE (Canon Medical Systems, Otawara, Japan) was used, and the imaging conditions were as follows: slice thickness, 2.0 mm; tube voltage, 120 kV; and 110 mAs. RayStation 10A (RaySearch Laboratories, Stockholm, Sweden) was used for treatment planning. The therapeutic carbon ion beam was used in the irradiation experiments. A 5.0 × 5.0 × 5.0 cm VOI was created from the phantom surface and centered on the beam incidence point. The treatment plan was performed to deliver a uniform physical dose to the VOI. Therefore, a VOI covering a 5.0 × 5.0 × 5.0 cm target area was created, and a treatment plan was developed to ensure a uniform physical dose within the VOI. Figure 1d shows the calculated treatment plan dose distribution. The treatment plan was established, and dose calculations were performed with a pencil beam algorithm. The irradiation method was scanning irradiation using the full energy scanning method.^24^ Dose and particle range calculations were performed using the stopping power ratio calculated from the CT values of the phantom. We also made additional measurements using an ionization chamber. The geometry of the measurement is shown in Figure 2a. The PTW type 34001 Roos ionization chamber (PTW, Freiburg GmbH, Freiburg, Germany) was used as the ionization chamber, and Tough Water was used as the phantom. The irradiated treatment plan was the same as the film measurement, and the physical dose was uniform within a 5.0 × 5.0 × 5.0 cm VOI target.

### 2.3 Evaluation

Irradiation to the phantom was performed at 2.0 Gy, both with and without the stent sandwiched in the phantom. In addition, for cases without the stent, irradiation was also conducted at 1.8 and 2.2 Gy. The evaluation was performed using the EBT4 film, specifically by analyzing the average pixel value within a 2.0 × 2.0 cm area at the center of each irradiated field. The physical doses within the targets set 1.8, 2.0, and 2.2 Gy. In addition, each of the five different stents was irradiated at a dose of 2.0 Gy in between the phantoms. Film measurements at each depth under each condition were evaluated. The film scanner was EPSON Offirio ES‐10000G (Seiko EPSON, Suwa, Japan), and the scanning software was Film Scan New (R‐TEC. Inc., Tokyo, Japan). The red channel extracted from the RGB transmission images was used for analysis. For all stents, similar measurements were performed twice each, and the average of the measurements was evaluated. Film scans were performed three times for each film, and the evaluation was based on the average of the results obtained from the two measurements. The scanned images were evaluated in Image J (https://imagej.net/contribute/citing) by extracting the average pixel values in the center of a 2.0 × 2.0 cm area. In the measurements with the ionization chamber, the measurements without stents at 1.8, 2.0, and 2.2 Gy were compared with those obtained when the five stents were placed in front of the ionization chamber and irradiated with 2.0 Gy. All measurements were normalized by the mean value of the 2.0 Gy measurements without a stent.

## 3 RESULTS

**FIGURE 3:**
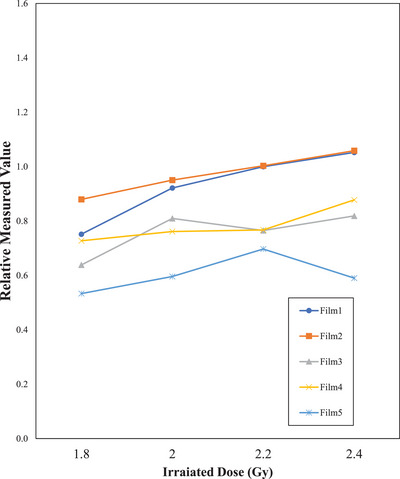
Relative pixel value of irradiation dose vs. film.

**FIGURE 4:**
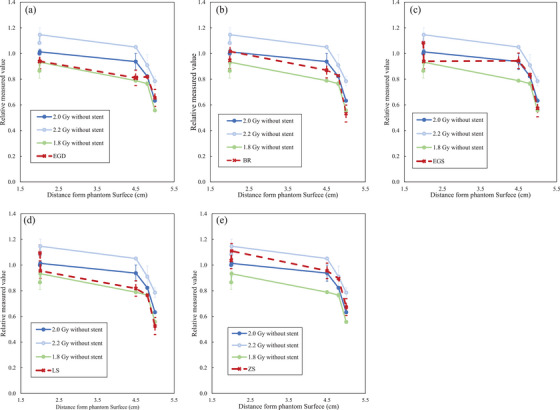
Relative film pixel value with a stent in place. The measurement results when (a) EGS, (b) BR, (c) EGS, (d) LS, and (e) ZS were placed. BR, BileRush Selective; EGD, EGIS biliary stent double bear; EGS, EGIS biliary stent single bear covered; LS, Niti‐S Less Shortening D‐type Stent; ZS, ZEO Stent V.

**FIGURE 5:**
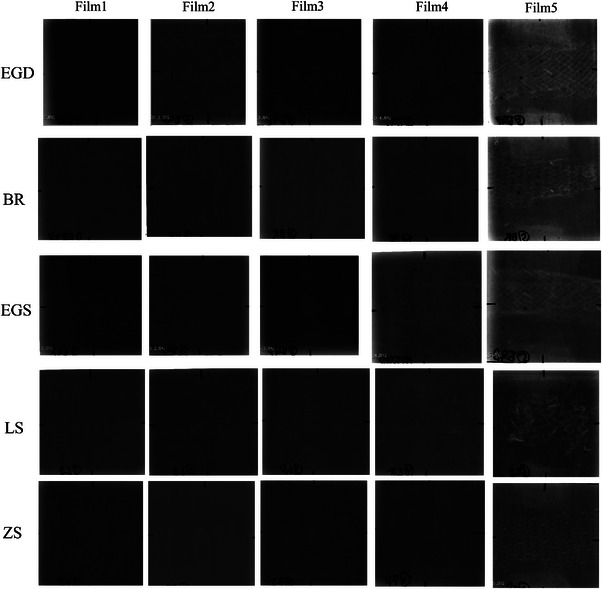
Example images of films after irradiation. BR, BileRush Selective; EGD, EGIS biliary stent double bear; EGS, EGIS biliary stent single bear covered; LS, Niti‐S Less Shortening D‐type Stent; ZS, ZEO Stent V.

Figure 3 shows the relationship between irradiation dose and EBT4 film pixel value. The pixel value was found to be generally linear for all films. As for the linearity evaluation, R^2^ for film1‐5 were 0.929, 0.995, 0.596, 0.817, and 0.265, respectively. Figure 4a–e shows film measurements with EGS, BR, EGS, LS, and ZS stents in place and 1.8, 2.0, and 2.2 Gy without stents, respectively. Film measurements at all depths, with any stent, were located inside the 1.8 and 2.2 Gy measurement lines and were close to the 2.0 Gy measurement line. The measured values of the film at the distal end of the range were lower than those of the film at the center of the VOI. The variation of values within the 2.0 cm × 2.0 cm evaluation area was < 2% relative to the film1 value without a stent of 2 Gy for all measurements. Figure 5 presents the images of the films at five measured depths after carbon‐beam irradiation across each stent. The shading of the stent shape was observed in film 5, which was located closest to the distal end of the VOI. In the film measured across the Niti‐S Less Shortening D‐type Stent, film 4 also showed film shading that might have been caused by the stent. The results of the ionization chamber measurements are shown in Figure 2b. Without stent, measurements at 1.8 Gy and 2.2 Gy irradiation were ± 10% of the 2.0 Gy irradiation measurements, respectively. In contrast, the measurements at 2.0 Gy irradiation with stent were in <1.5% agreement with measurements with 2.0 Gy without stent.

## 4 DISCUSSION

This study evaluated the impact of metal bile duct stents on CIRT dose. Results showed that the dose change relative to the prescribed dose with a stent compared with that without a stent was <10%. Chen et al. showed that the dose‐increasing effect of metal stents in x‐ray therapy was negligible under multiple‐field irradiation conditions.^22^ Based on these reports, the effects of dose changes may be negligible with multi‐port irradiation. However, since the reduction of error by multi‐port irradiation was not verified in this study, further verification by Monte Carlo simulation or other methods would support the possibility of clinical application.

Kuwatani et al. found that the recurrence rates of biliary obstruction in patients with pancreatic cancer were 6% in the metal stent group and 83% in the plastic stent group.^9^ Hence, the recurrence rate of biliary obstruction in patients with metal stents was significantly lower than that in those with plastic stents. Similarly, Kobayashi et al. found that the reintervention rates after metal stent insertion were significantly lower than those after plastic stent insertion.^25^ A previous study has reported that the need for reintervention with metal stents was significantly lower than that with plastic stents. This indicates less physical, emotional, and financial burden for the patient and medical staff. Therefore, the use of a metal stent in cases of pancreatic cancer requiring bile duct drainage is significantly advantageous, and this may also be true in patients receiving particle therapy. This study evaluated the impact of metal stents in CIRT, and the results support the application of CIRT in patients with metal stents.

Li et al. reported changes in dose distribution around the stent during x‐ray therapy.^26^ Moreover, they reported that the maximum dose perturbation in the region 0.5–4.0 mm from the stent was approximately 10%. Jalaj et al. evaluated the effect of metal stents on dose perturbations in proton therapy. Results showed that the dose perturbation ranged from 4% to 11% on both the proximal and distal sides of the beam. Their results are similar to those of the current study, which indicated that the effect of metallic stents on dose perturbations is comparable to that on carbon ion beams.

Several studies have evaluated the impact of internal body metals on CIRT dose distribution. Reidel et al. investigated the effect of several markers on the carbon ion beam and found a dose impact of approximately 3%–5%.^27^ Tsubouchi et al. examined the effect rate of the carbon ion beam with gold markers, and it was found to be <10%.^28^ This result is generally similar to those of the current study. Our results indicate that the dose perturbation due to the stent was <10%, as shown in Figure 4a–e, since the measurements with the stent were located within the range of <10% of the measurements without the stent. In addition, the results of the ionization chamber measurements (Figure 2b) showed only <1.5% change in the with‐stent situation compared to the without‐stent situation. However, there might be differences in materials, and the effects of metals on carbon ion beams are similar to those of markers and stents. Therefore, the effect rate of a typically used marker or stent‐sized metal on carbon ion beams is approximately <10%. Nevertheless, a more detailed investigation should be conducted to identify a more accurate treatment.

Figure 4 shows that there was a slight decrease in dose from film 1 to film 3. The response of the EBT4 films to the carbon ion beam is not clear, but the variation in measurements could be due to linear energy transfer or particle energy dependence. Therefore, the evaluation of the dose impact of the stent was based on a comparison of film measurements at the same depth. The poor linearity of the response to dose in film 5 may be due to the uncertainty in the particle range at the end of the dose distribution. Although film 5 was set to be within the VOI, a significant dose reduction was observed relative to the measured values of films at other locations. This may be due to the thickness of the film and stent. The total thickness of the five films and the stent is assumed to be several millimeters, and the dose reduction area outside the VOI is considered to have been measured.

In this study, the experimental system was simplified by flattening the stent. This should allow us to validate the results of this study compared to a Monte Carlo simulation with a simple geometry. In actual treatment, stents have a three‐dimensional structure, so the impact of metal stents may differ from the results of this study. More detailed studies are needed, and based on the results of this study, experiments, and Monte Carlo simulations that replicate actual treatments are important.

In this study, we did not experiment with plastic stents. Although plastic stents do not have exactly the same stopping power ratio as biological materials, they are expected to have less impact on the treatment plan than metal stents. In fact, carbon ion therapy for plastic stent insertion cases is acceptable.^5^ Therefore, it is worthwhile to compare metal and plastic stents in the evaluation of their impact on therapy.

Materials such as plastics and metals that are not considered in treatment planning can have a significant impact on particle therapy. In particular, even small metallic materials can cause significant treatment errors if they are not properly considered in treatment planning. Stent shape dose reduction was observed at the distal end of the VOI. This is attributed to the fact that the stent is metal, which can change the range of the carbon ion beam passing through the stent. Based on this result, a dose‐lowering region is established at the distal end of the particle range in accordance with the shape of the stent. Moreover, the extent of dose reduction differed based on the type of stent. Differences in stent construction and materials might have influenced the results. Four of the five stents examined in this study showed dose reduction at film 5 at the end of the VOI. However, dose reduction was not observed at film 4, which has a 2 mm shallower depth from the end. The effect of the stent on the range was approximately ≤2 mm.

This study evaluated macroscopic dose changes. However, the detailed effects of the fine geometry of the stent were not examined. This study is based on a simple presentation of data for a basic study and is a stepping stone for future detailed studies. Given that this is only a basic study, further detailed studies are essential for clinical application. Therefore, further studies must be performed to validate the impact of the metal stent in more detail using Monte Carlo simulations or other methods based on the results of the current study. In addition, this study only evaluated dose changes. The actual development of adverse events is attributed to not only the notional change in dose but also factors such as mechanical dilation induced by the stent and its interaction with radiation. Based on the results of this study alone, CIRT cannot be recommended to patients with metal stents. Future clinical applications should be fully discussed in light of the reported impact of body metals on adverse events after radiotherapy. In this study, the validation was performed in a relatively shallow area of 5 cm from the phantom surface. Carbon ion therapy is also used to treat tumors in deeper locations. In this case, carbon ion beams with higher kinetic energy are used. Therefore, studies using higher particle beam energies are important to reproduce the clinical situation. Although the characteristics of the response of EBT4 films in x‐ray therapy have been reported,^29^, ^30^, ^31^ their characteristics for particle beams are not clear. In this study, we only evaluated the relative values between the presence and absence of metal stents. For example, future research on absolute dosimetry should characterize the response of EBT4 to particle beam.

## 5 CONCLUSION

In conclusion, this study evaluated the effect of bile duct metal stents on CIRT dose and found that the effect rate of the metal stent on the dose was <10%. The dose perturbation was comparable to that of x‐rays and proton beams, which have been reported previously. Based on the basic studies in this study, it is desirable to further evaluate the impact of metal stents in carbon beam therapy in more detail through Monte Carlo simulations and experiments that reproduce clinical situations.

## AUTHOR CONTRIBUTIONS

Yuya Miyasaka: Conducting the experiment, writing the manuscript, and data analysis. Tetsuya Ishizawa and Yoshihito Nawa: Study design, clinical integration, clinical review, and review manuscript. Hikaru Souda: Conducting the experiment, data analysis, and review the manuscript. Shohei Kawashiro and Hiraku Sato: Clinical integration, clinical review, and review manuscript. Hongbo Chai: Review data and manuscript review. Miyu Ishizawa: Review data and review the manuscript. Takeo Iwai: Management and coordination responsibility for the research activity planning and execution.

## CONFLICT OF INTEREST STATEMENT

The authors declare no conflicts of interest.

## Data Availability

Research data are stored in an institutional repository and will be shared upon request to the corresponding author.
